# Smartphone-based activity research: methodology and key insights

**DOI:** 10.3389/fsurg.2025.1613915

**Published:** 2025-08-12

**Authors:** Ryan W. Turlip, Daksh Chauhan, Hasan S. Ahmad, Mert Marcel Dagli, Bonnie Y. Hu, Richard J. Chung, Yohannes Ghenbot, Ben J. Gu, Nisarg Patel, Richelle J. Kim, Julia Kincaid, Akash Verma, Jang W. Yoon

**Affiliations:** ^1^ Department of Neurosurgery, Perelman School of Medicine, University of Pennsylvania, Philadelphia, PA, United States; ^2^ Department of Neurosurgery, School of Medicine, University of Virginia, Charlottesville, VA, United States; ^3^ Department of Neurosurgery, Lewis Katz School of Medicine, Temple University, Philadelphia, PA, United States; ^4^ Department of Neurosurgery, Columbia College, Columbia University, New York, NY, United States

**Keywords:** accelerometer, activity tracking, big data, biometrics, smartphone

## Abstract

**Background and objectives:**

Objectively studying patient outcomes following surgery has been an important aspect of evidence-based medicine. The current gold-standard—patient reported outcomes measures—provides valuable information but have subjective biases. Smartphones, which passively collect data on physical activity such as daily steps, may provide objective and valuable insight into patient recovery and functional status. This study aims to provide a methodological guide for data collection and analysis of smartphone accelerometer data to assess clinical outcomes following surgery.

**Methods:**

Patient health metrics—namely daily steps, distance travelled, and flights climbed—were extracted from patient smartphones using easy-to-download applications. These applications upload the data that smartphone accelerometers passively collect daily to a HIPAA compliant encrypted server while de-identifying the patient's personal health information. Patients were consented in multiple settings—synchronously during clinical visits or asynchronously over the phone—and could be enrolled during the initial pre-operative visit or well after the surgery. With the patient data acquired, the peri-operative window of selection is determined based on the needs to the study. The timeseries data is then statistically normalized to account for individual baselines and smoothened over a 14-day moving average to minimize noise. Mathematical analysis can be harnessed to study quantifiable recovery and decline periods, which provide continuous and nuanced insight into patient's health throughout their spine disease and treatment course. Additionally, integrating clinical variables permits computational machine models capable of predicting patient trajectories and guiding clinical decisioning.

**Conclusion:**

Smartphones offer a new metric for studying patient well-being and outcomes after surgery. The research with them is in its nascent stages but further studies can potentially revolutionize our understanding of spinal disease.

## 1 Introduction

Objectively studying patient well-being and perioperative outcomes has been the cornerstone of evidence-based medicine that has guided policy making and standard of care for the past few decades (1). Neurosurgeons, especially spine specialists, have harnessed patient-reported outcome measures (PROMs) such as EQ-5D, a measure of quality of living, and Oswestry Disability Index (ODI), a measure of disability, to understand patient well-being (2, 3). However, these survey-based metrics have certain disadvantages such as their discrete nature, subjectivity, patient recall bias, and the intra-administration variability (4, 5). These drawbacks make PROMs suboptimal for capturing the varied clinical progress that patients with complex neurological conditions, such as vestibular schwannomas, demonstrate over the course of their recovery (6, 7). Similarly, in multi-level spondylolisthesis, functional gains may continue despite PROMs reaching a ceiling, missing important aspects of recovery (8). There is a need for new metrics that capture patient well-being more objectively and on a more minute scale.

Smartphones can capture patient daily activity with high-fidelity accelerometers. Physicians and scientists have investigated this new modality to decode a patient's physical activity outcomes over the past decade (9, 10). Recent literature harnessing smartphone-captured steps and mobility datapoints have demonstrated that these up-and-coming mobility variables can quantify and describe patient well-being following surgery (11–14). For instance, Ahmad et al. have highlighted how patient declines and recoveries can be studied in a data-driven manner and quantified in discrete epochs correlating with clinical worsening or improvement of pathologies (15). Other papers have further validated smartphone-based metrics by demonstrating their correlation with pain-related PROMs such as VAS Pain and PROMIS Pain Interference scores (16). Chauhan et al. also highlighted the value of smartphone-captured steps for differentiating recovery trajectories between lumbar fusion and lumbar decompression patients—a step toward identifying distinct recovery phenotypes, or patterns of functional improvement (17).

Mobility measures are particularly useful to measure in patients presenting with spine pathologies and movement disorders, which can present with complex pre-operative and post-operative courses. For example, while higher physical activity following lumbar spine surgery is associated with improved outcomes (18), it is also associated with increased pain (19, 20), something that data-driven phenotyping may potentially explain. This nuanced picture of patient activity provided by smartphones, when used simultaneously with the existing PROMs, provides a promising avenue to elucidate what contributes to desirable outcomes following surgery.

Most neurosurgical research harnessing smartphone-captured metrics so far has been spine-focused. Future work should expand not only to include broader patient populations but also to capture patients of other subspecialties within and outside of neurosurgery. Our group initiated the work on smartphone-captured accelerometry at a large academic center in 2020 and has since executed multiple studies and analyses. In this manuscript, we aim to share our experience and the methodologic steps for executing the studies harnessing patient smartphone-captured physical activity metrics.

## 2 Methods

This manuscript is structured as a descriptive methods paper intended to guide researchers in implementing smartphone-based accelerometry for clinical outcomes research.

### 2.1 Institutional approval and informed consent

Acquisition of patient smartphone accelerometer data and informed consent was approved by the Institutional Review Board (IRB #843229) on 12/02/2021. Data collection began in December 2021 and is ongoing as part of an active, longitudinal research initiative.

### 2.2 Data acquisition

#### 2.2.1 Patient populations

The methodology described in this paper has been applied across a variety of neurosurgical patient populations, including individuals undergoing lumbar fusion for degenerative spine disease, cervical spine procedures, peripheral nerve surgery, and craniotomies. While most of our prior analyses have focused on patients with lumbar degeneration, the data acquisition pipeline and software infrastructure are generalizable across clinical subspecialties. In individual outcome studies applying this framework, patients are selected and stratified based on clinical presentation (e.g., neurogenic claudication, lumbar radiculopathy), with strict inclusion/exclusion criteria to ensure comparability across surgical cohorts. In general, eligibility criteria for participation included patients aged ≥18 years, fluent in English, in possession of an Apple iPhone, and able to provide informed consent for data sharing. Specific inclusion or exclusion criteria may vary slightly by study focus and are defined in individual project protocols.

#### 2.2.2 Software

Relevant health metrics from each patient's smartphone were extracted using an application such as Kinesiometrics (Kinesiometrics Inc., Miami, FL, USA) or QS Access (Quantified Self Labs, San Francisco, CA, USA), which accesses stored health information. Such applications access a smartphone's integrated health application programming interface (API) which stores health metrics. Our studies limited enrollment to patients with Apple iPhones (Apple Inc., Cupertino, CA, USA) since, unlike other smartphones, the Apple HealthKit API retrospectively stores health data, enabling retrospective data collection. For example, iPhones typically collect health information using accelerometers starting the day of purchase, yielding substantial pre-operative and post-operative time points for patients. Additionally, mobility data, such as steps-per-day, is recorded hourly, providing highly granular and objective information pertaining to the patient's functional recovery. Upon downloading the application, the patient elects to allow the health points stored on their smartphone to be shared to an encrypted HIPAA compliant server that removes identifying information and assigns a unique identifier number to their record. Each time a patient signs in to the application thereafter, updated health information is returned to the server, enabling prospective study enrollment and data collection.

#### 2.2.3 Asynchronous data collection

**Figure 1:**
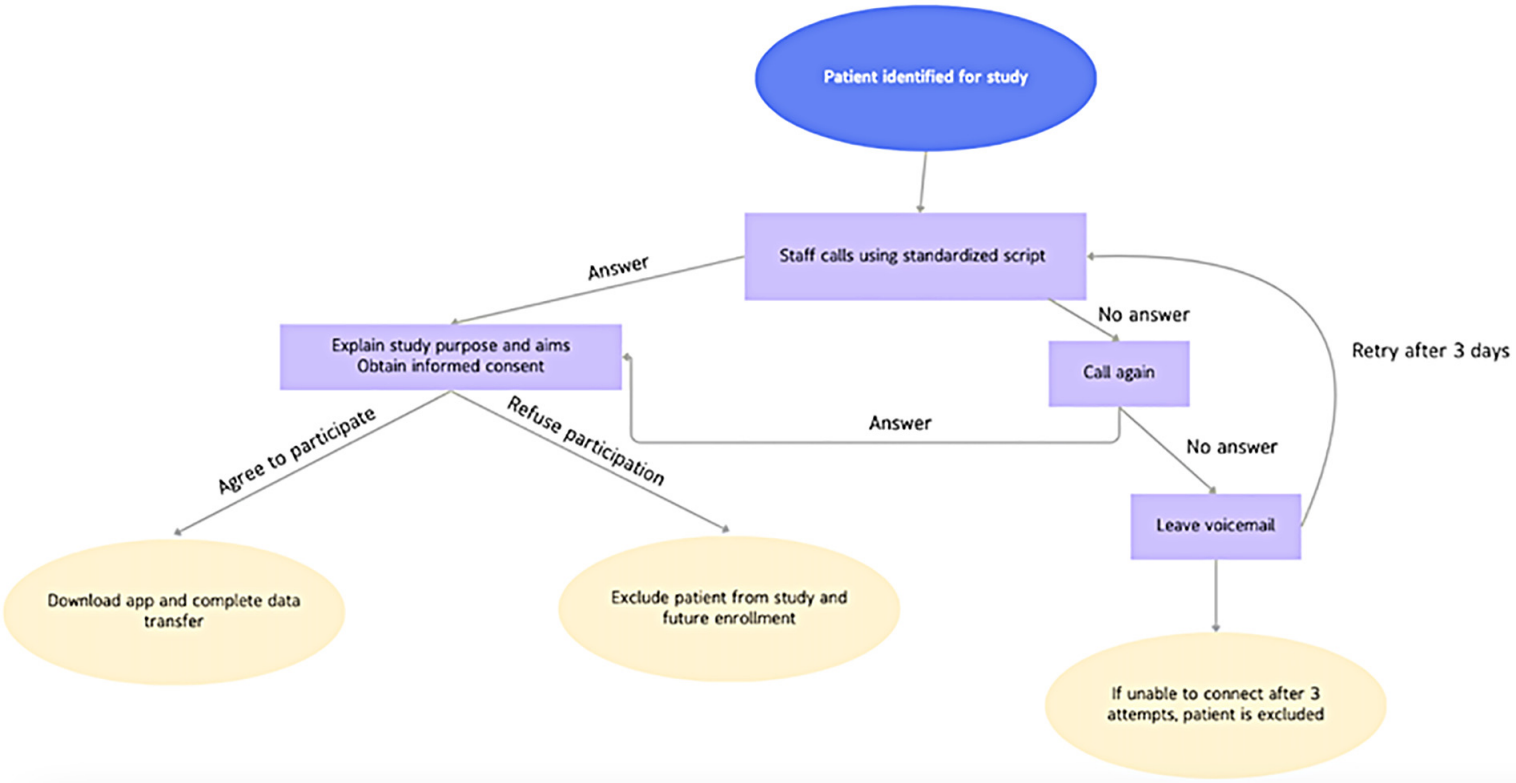
Synthesis of data collection workflow.

Patient consent and data acquisition can be acquired using 2 main methods: asynchronous patient calling and consent, or synchronous patient enrollment during perioperative clinical visits. Asynchronous enrollment requires institutionally trained staff to individually call each eligible patient to receive informed consent. Research assistants, such as an undergraduate or medical students, are given a standardized script approved by the Institutional Review Board, first explaining the study purpose and aims. After obtaining consent, the patients are guided through downloading the approved software to their smartphones, creating a unique account, and enabling transfer of stored accelerometer data. If a research assistant fails to connect with the patient upon first calling attempt, a voicemail message is left with a return number. If the patient cannot be reached on three different occasions, the patient is excluded from the study (Figure 1).

In a typical recruitment cycle, all neurosurgical patients who were at least one-year post-operative were eligible for outreach. Of the eligible pool, 10%–20% of patients who were contacted were both successfully reached and consented, which is in accordance with previous studies (15, 21). Reasons for exclusion included inability to reach the patient after three call attempts, patient refusal to consent to data transfer, or lack of usable smartphone accelerometer data (e.g., insufficient historical data or HealthKit not enabled).

#### 2.2.4 Synchronous data collection

Patients can also be consented to participate prospectively or retrospectively in the study through synchronous enrollment during clinical encounters. For retrospective enrollment, when a patient is returning for a post-operative visit, a research assistant can accompany the physician conducting the visit and aid the patient in downloading the application and transferring health data at the conclusion of the clinical visit. A QR code is used to easily direct the patient's smartphone to the application download on the app store. Depending on the length of time since surgery, this step alone can yield adequate post-operative time points for further analyses. For patients undergoing surgical intervention, enrollment can be done prospectively prior to their surgery, and the application, once downloaded on patient smartphones, can continue to upload data to the institutional serve post-operatively.

### 2.3 Post-acquisition pre-processing

Once patient data is acquired, researchers can evaluate key mobility metrics such as daily steps, flights of stairs climbed, and total distance traveled, typically from an exported spreadsheet. These metrics are considered in relation to the surgery date, so defining the peri-operative window is essential and should align with the study's aims. While most studies examine a period of at least one year before and one year after surgery, this timeframe may be adjusted based on the specific project requirements. After selecting the analysis window, patient data is statistically normalized to account for individual baselines and smoothed over a 14-day moving average to minimize noise and identify true trends. For higher-resolution studies, a shorter smoothing window may be used, though this can introduce additional noise. This moving average technique attempts to detect short-term signal artifacts, reduce outliers, and remove erroneous measurements, and it has been well-described in similar applications across various medical specialties (22–24). This normalization is critical for cross-patient comparisons, as it adjusts for natural baseline differences. For instance, a sedentary patient averaging 4,000 steps daily would experience a more significant impact from a 1,000-step increase post-surgery than a highly active patient averaging 12,000 steps. Employing normalized data frames that highlight mobility changes in terms of standard deviations allows researchers to mitigate baseline discrepancies and better compare outcomes. To ensure adequate data quality, we included only patients with at least one year of pre-operative and one year of post-operative activity data. Furthermore, any patient-months with more than 20% missing or zero-activity days were excluded from analysis to minimize bias from incomplete data capture.

### 2.4 Analytics pipeline

Following acquisition and preprocessing, the analytical potential of the data is vast and adaptable to the study's objectives. Even a single patient with a year's worth of pre- and post-surgical activity data yields 730 data points, forming a rich, longitudinal mobility profile. Analyzing such extensive datasets requires robust computational tools and programming to derive insights efficiently.

In our analytical workflows, we have utilized both quantitative and qualitative methods. For example, in Ahmad et al., we used pooled, data-driven approaches to segment perioperative mobility into five distinct “epochs”: preoperative baseline, acute preoperative decline, immediate postoperative recovery, full recovery, and secondary decline (15). Thresholds for activity changes—measured in standard deviations—were established to quantify recovery and decline periods, which were then mapped alongside clinical symptom timelines to provide a detailed view of patient trajectories. These stages were validated against clinical documentation in 92% of patients, demonstrating a strong correlation between mobility-derived and clinician-documented recovery trajectories. Notably, 79% of patients ultimately achieved full recovery, defined as a sustained 80% improvement in activity above their preoperative baseline (*P* = .002). Similarly, in Chauhan et al., we compared two surgical approaches—lumbar fusion and lumbar decompression—by examining the duration and rate of activity changes within these epochs (17). Activity data revealed significant differences in both recovery duration and total improvement between the groups, highlighting how continuous mobility metrics can capture nuanced differences in real-world functional outcomes. For example, decompression patients tended to show faster return to baseline activity, while fusion patients had a slower but more sustained increase in steps over time.

**Figure 2:**
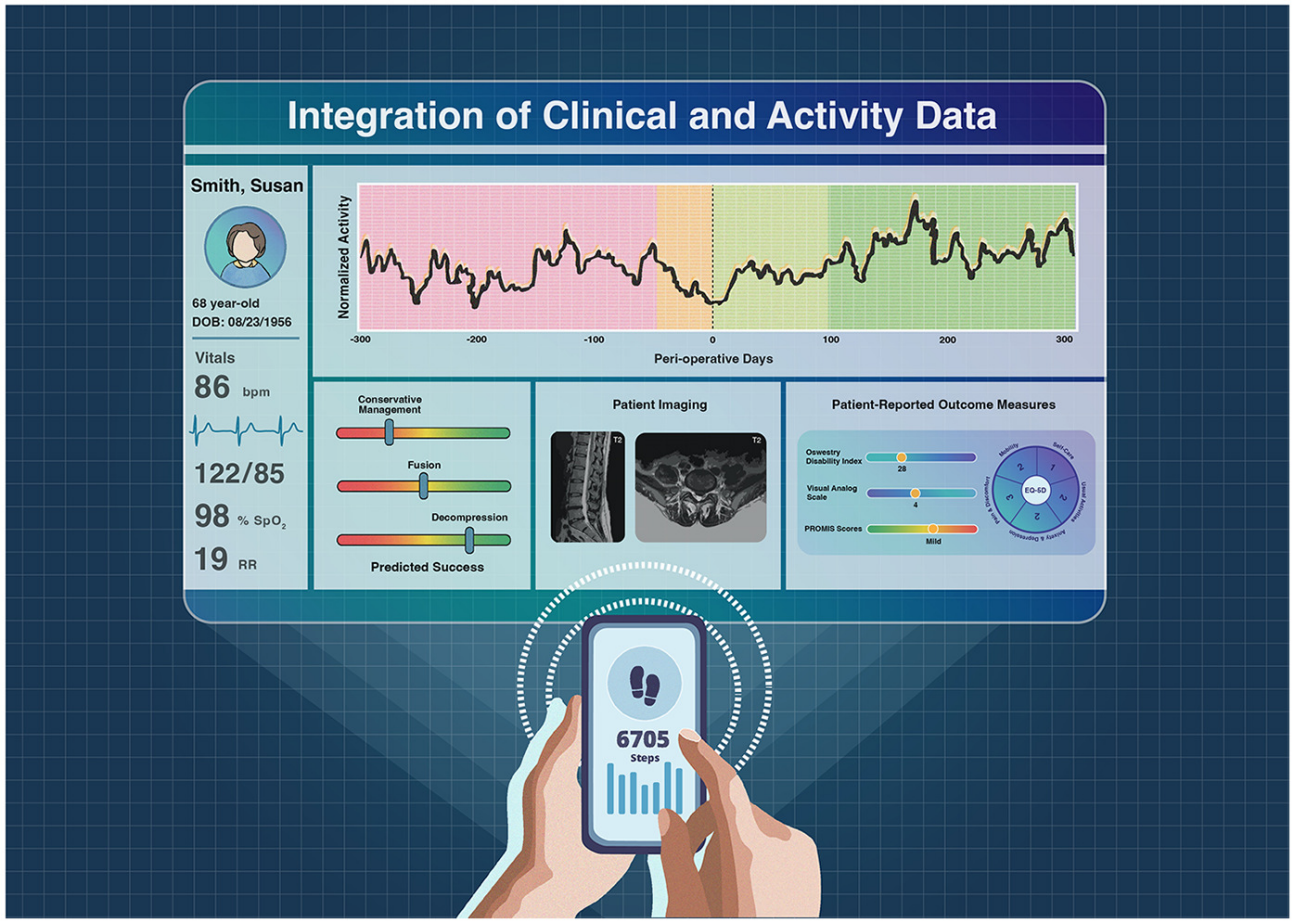
Dashboard integrating PROMs and patient activity data into electronic health record.

Additionally, we integrated clinical variables such as age, BMI, and Charlson Comorbidity Index with quantitative mobility data to create supervised machine learning models—including logistic regression, random forest, and XGBoost—capable of predicting the likelihood of a patient experiencing decline after an initial recovery phase (25). These models incorporated features such as recovery duration, activity trends, age, and BMI, with the random forest model achieving the highest predictive accuracy (86.7%). While defining recovery and decline in discrete epochs provides a useful framework for assessing patient outcomes, treating activity data as a continuous stream has distinct advantages. Continuous metrics, such as the proportion of the post-operative period during which a patient exceeds pre-operative baseline activity levels, offer a less biased and more comprehensive view of recovery compared to epoch-based methods. This continuous approach is particularly valuable for comparing multiple patient cohorts, such as socioeconomic quintiles (26), in studies focused on healthcare disparities, as it captures subtle shifts and patterns in patient mobility without being limited by arbitrary categorizations (Figure 2).

## 3 Discussion

The present methodological manuscript examines the utility and potential of smartphone accelerometer data in assessing mobility outcomes for spine surgery patients. By leveraging routinely collected, passively monitored data such as daily step counts, our methodology seeks to address some limitations inherent in traditional PROMs, including subjectivity, recall bias, and discrete data collection intervals (27). The use of continuous, clinically meaningful mobility metrics presents a promising path forward for understanding post-surgical recovery trajectories, with the potential to refine patient phenotyping and treatment personalization.

Prior work has attempted to categorize patient outcomes according to PROMs following spine surgery (28, 29), but our experience aligns with emerging efforts to expand beyond conventional PROMs. Previous studies have demonstrated that significant events in a patient's recovery can be identified through activity data (9–11, 30). These studies reveal patterns of decline and recovery that align with clinical symptoms, supporting the idea that smartphone-based metrics can serve as reliable proxies for assessing patient progress. Further work validates the approach by illustrating that recovery trends differ between surgical procedures, such as lumbar fusion vs. lumbar decompression, showcasing the granularity that smartphone data can provide for comparative effectiveness research (17). Future efforts will focus on validating smartphone-derived activity metrics against conventional recovery endpoints such as PROMs, timed functional tests, and return-to-activity milestones to further establish their clinical utility.

While our methodology harnessed passively collected health data that is stored retrospectively, other groups have analyzed biometric information through wearable devices. One group used Apple Watches (Apple Inc., Cupertino, CA) on 30 cervical spondylosis patients to compare their functional activity before and after surgical intervention (30). The same team used the Apple Watch to estimate physical activity leading up to elective surgery for degenerative spine disease (31). Another group used a smartphone application to automate PROMs collection post-operatively to actively track outcomes and reduce additional visits (32). Although wearables may be even more accurate at measuring patient physical activity than smartphones, they are costlier to harness and post a greater barrier to entry than using smartphones, which are more widely used.

### 3.1 Unlocking the future of mobility metrics and big data in medicine

Smartphone accelerometry data is emerging as a valuable tool for uncovering associations between surgical procedures and their outcomes. The extensive, longitudinal activity data captured by smart devices enables the creation of statistical and predictive models by leveraging high-fidelity data across substantial time periods. The concept of “big data”—utilizing large datasets to identify meaningful mathematical patterns—is rapidly becoming a powerful approach for clinical prognostication and decision-making (33). Unlike traditional measures that provide infrequent snapshots of a patient's recovery stages, objective activity data provides significantly finer temporal resolution.

Furthermore, smartphone-based data collection is inherently scalable and could be applied across a wide range of patient populations and clinical contexts. The asynchronous data collection methods employed in this study streamline patient enrollment and data acquisition, making it feasible to implement in both high-resource academic settings and more constrained healthcare environments. As smartphone ownership is nearly universal and essential to many people's lives (34), this approach has the potential to bridge gaps in patient monitoring, providing a cost-effective, minimally intrusive means of tracking recovery even for patients in remote or underserved areas. This accessibility may help address disparities in healthcare outcomes by enabling continuous, standardized data collection across diverse demographic groups.

Future directions for this research involve extending this methodology to broader patient populations, integrating accelerometer data with other clinically meaningful health metrics, and incorporating machine learning techniques to enhance predictive capabilities. For example, applying machine learning models to these data could help identify patients at higher risk of postoperative decline, thereby allowing for targeted interventions. Ultimately, we believe that the future of spine surgery outcomes will use functional mobility data in tandem with traditional PROMs, and correlations between smartphone-based mobility and VAS have already been reported (16). Studies involving non-spine populations, as well as non-surgical cohorts, could also reveal further applications, such as in chronic pain management or other movement disorders, broadening the relevance of smartphone-based metrics in neurosurgery and beyond.

### 3.2 Barriers to adoption: technological, logistical, and data integration challenges

Despite its advantages, smartphone-based data collection presents notable challenges that must be addressed to enable wider adoption. One limitation of this study is its reliance on Apple iPhones, which reduces generalizability to patients who use other devices. However, retrospective data is not stored on the Android Health Platform API, so Android users can only be enrolled in prospective studies. This platform-specific approach excludes Android users and others, potentially creating biases in patient sampling. Expanding the compatibility of data collection tools across platforms is necessary to ensure more representative datasets and scalability. Further, variability in smartphone use and patient engagement can affect data quality. Patients may alter their behavior when aware their activity is being monitored, introducing a potential Hawthorne effect that could skew results for prospective studies. Additionally, this method relies on consistent smartphone usage, which may be less applicable for older populations or individuals unfamiliar with smartphone technology.

Managing a centralized database for smartphone-based mobility data also comes with institutional, legal, and financial challenges. From an institutional standpoint, maintaining such a repository requires significant investment in secure server infrastructure and skilled personnel to oversee data handling and compliance. Legally, these systems must adhere to stringent regulations, such as HIPAA, to protect patient privacy and prevent data breaches. The encryption and de-identification processes, while necessary, add layers of complexity to data management. Financially, these efforts require ongoing funding, not just for infrastructure and staff but also for licensing software and maintaining interoperability with evolving smartphone platforms.

Another practical challenge lies in obtaining informed consent and enrolling patients. This process often involves individualized outreach, including phone calls to guide patients through the consent process and app installation. Such methods are labor-intensive and time-sensitive, particularly in asynchronous enrollment scenarios, where the inability to connect with patients may result in incomplete participation. Streamlining this process through automated or simplified workflows could improve recruitment rates and reduce resource burdens.

A further consideration is the integration of smartphone mobility data with clinical data, such as electronic health records (EHRs). Aligning these disparate datasets to ensure accuracy and consistency can be time-intensive and logistically complex. The effort required to synchronize activity metrics with detailed clinical variables may delay analyses or require substantial institutional support. However, this integration remains crucial for contextualizing mobility data within the broader framework of patient health and surgical outcomes.

## 4 Conclusion

The present work illustrates a practical framework for conducting smartphone accelerometer-base mobility research and its power to complement and even enhance traditional outcome measures. By leveraging familiar technology in innovative ways, we can advance our understanding of recovery patterns and improve patient outcomes. As our understanding of objective patient metrics expands, integrating such data into routine clinical practice could transform patient monitoring, providing insights that are both actionable and personalized. By sharing our methodology and the insights gained from our experience, we hope to encourage further research and wider adoption of smartphone-based patient monitoring, ultimately contributing to a more comprehensive and individualized approach to patient care in spine surgery.

## Data Availability

The original contributions presented in the study are included in the article/Supplementary Material, further inquiries can be directed to the corresponding author.
